# Is a Washer a Mandatory Component in Young Trauma Patients with S1-S2 Iliosacral Screw Fixation of Posterior Pelvis Ring Injuries? A Biomechanical Study

**DOI:** 10.3390/medicina59081379

**Published:** 2023-07-28

**Authors:** Till Berk, Ivan Zderic, Peter Schwarzenberg, Torsten Pastor, Sascha Halvachizadeh, Geoff Richards, Boyko Gueorguiev, Hans-Christoph Pape

**Affiliations:** 1 AO Research Institute Davos, Clavadelerstrasse 8, 7270 Davos, Switzerland; 2 Department of Trauma, University Hospital Zurich, Raemistrasse 100, 8091 Zurich, Switzerlandhans-christoph.pape@usz.ch (H.-C.P.); 3 Harald-Tscherne Laboratory for Orthopedic and Trauma Research, University of Zurich, Sternwartstrasse 14, 8091 Zurich, Switzerland; 4 Department of Orthopaedic and Trauma Surgery, Cantonal Hospital Lucerne, 6000 Lucerne, Switzerland

**Keywords:** biomechanics, motion tracking, posterior pelvis ring injury, S1-S2 sacroiliac screw fixation, synthetic pelvic bone model, transsacral fixation, washer, washerless

## Abstract

*Background and purpose*: Cannulated screws are standard implants for percutaneous fixa-tion of posterior pelvis ring injuries. The choice of whether to use these screws in combination with a washer is still undecided. The aim of this study was to evaluate the biomechanical competence of S1-S2 sacroiliac (SI) screw fixation with and without using a washer across three different screw designs. *Material and Methods*: Twenty-four composite pelvises were used and an SI joint injury type APC III according to the Young and Burgess classification was simulated. Fixation of the posterior pelvis ring was performed using either partially threaded short screws, fully threaded short screws, or fully threaded long transsacral screws. Biomechanical testing was performed under progressively increasing cyclic loading until failure, with monitoring of the intersegmental and bone-implant movements via motion tracking. *Results*: The number of cycles to failure and the corresponding load at failure (N) were significantly higher for the fully threaded short screws with a washer (3972 ± 600/398.6 ± 30.0) versus its counterpart without a washer (2993 ± 527/349.7 ± 26.4), *p* = 0.026. In contrast, these two parameters did not reveal any significant differences when comparing fixations with and without a washer using either partially threaded short of fully threaded long transsacral screws, *p* ≥ 0.359. *Conclusions*: From a biomechanical perspective, a washer could be optional when using partially threaded short or fully threaded long transsacral S1-S2 screws for treatment of posterior pelvis ring injuries in young trauma patients. Yet, the omission of the washer in fully threaded short screws could lead to a significant diminished biomechanical stability.

## 1. Introduction

Cannulated screws are the gold standard for percutaneous treatment of sacroiliac (SI) pelvis ring injuries [1,2]. A combined fixation at both S1 and S2 levels via two SI screws provides the best type of fixation in case of a vertical pelvic instability [3,4]. Most common, screws of 6.5 mm or 7.3 mm diameter are used together with washers [5], the latter considered as advantageous regarding biomechanical fixation enhancement [6]. While there is a common agreement on the surgical treatment, the question of possible implant removal (IR) remains controversial. Literature provides extensive data on IR rates following SI screw fixation, amounting up to 57% in follow-up cohort studies [7]. Typical indications for IR are screw malposition, non-union, screw loosening, as well as iatrogenic damage to the L5 level or sacral nerve roots [8,9,10,11]. A complete IR might be indicated in cases with infection, allergic reactions, as well as unsettled pain or following an explicit patient request. However, complications following IR are common and range between 3 and 20% [12,13,14]. The fusion of the SI joint, especially in young and active patients, must be considered unphysiological. It was conversely stated that IR is justified in younger patients [7]. Some authors even recommend implant removal routinely [8,15]. Due to the difficulty and proneness to intraoperative problems, the retrieval of the washer can be the most demanding part of the surgery and constitute the longest part of the surgery [16,17]. Therefore, Oberst et al. suggested an endoscopic IR in order to reduce x-ray exposure and soft tissue damage [18]. Although young patients with predominantly physiological bone quality represent a clearly different patient clientele than geriatric patients, hardly any implants are specifically designed to patient’s age. Although there are surgical procedures and techniques that are adapted to the age of the patient, most implants are generic. Hence, young patients could potentially benefit from standard S1-S2 SI screw treatment excluding the washer, especially in view of IR, where surgery time, blood loss, surgical exposure, radiation exposure, and complications could be significantly lower. However, to our knowledge, the surgical method of S1-S2 SI screw fixation of the posterior pelvis ring without the washer has never been evaluated biomechanically.

### Purpose

The aim of this study was to investigate the biomechanical competence of S1-S2 SI screw fixations, with and without using a washer, across three different screw designs—(1) partially threaded, (2) fully threaded short, or (3) fully threaded long transsacral SI screws. The focus was to obtain a holistic picture of construct stability, including the one immediately after instrumentation, as well as the temporal stability decay during dynamic loading, together with the ultimate construct endurance. In addition, relative movements between the two bone segments, between the screw and the ilium, as well as between the screw tip and the sacrum were assessed. These parameters were specifically chosen given the known common clinical failure modes associated with SI screw fixation, namely screw cutout or loosening, resulting in eventual overall fixation failure [19,20].

## 2. Materials and Methods

Twenty-four composite pelvises (Model 4060^®^, Synbone, Zizers, Switzerland) were used. An SI joint injury type APC III according to the Young and Burgess classification was simulated in all specimens [21]. To induce this injury, the material of the symphysis as well as the SI joint was removed, resulting in a total instability.

**Figure 1:**
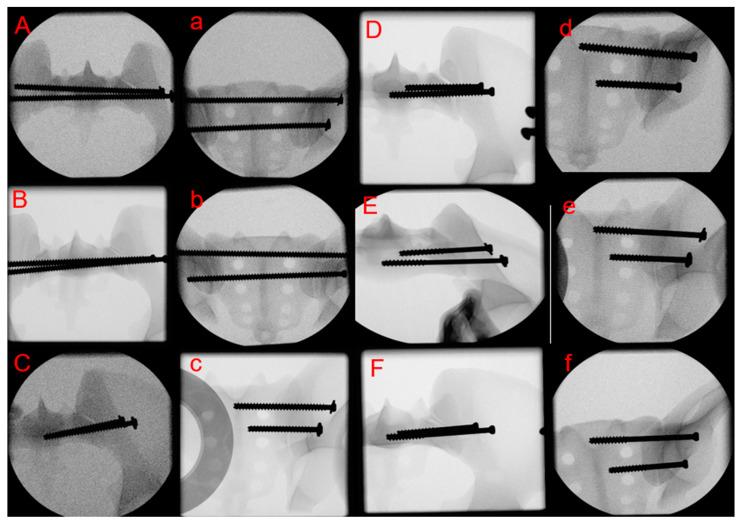
Inlet (uppercase letters) and outlet (lower case letters) x-rays after instrumentation showing specimens from groups TS w/(**A**,**a**), TS w/o (**B**,**b**), FT w/(**C**,**c**), FT w/o (**D**,**d**), PT w/(**E**,**e**) and PT w/o (**F**,**f**).

Specimens were stratified into three sets of two groups each for instrumentation as follows (Figure 1):

Set 1:

- Group PT w/: Fixation of the posterior pelvis ring with two 7.3 mm partially threaded short cannulated self-tapping screws (lengths: 90 mm for S1 and 65 mm for S2) and washers (diameter 13.0 mm, thickness 6.6 mm).
- Group PT w/o: Fixation of the posterior pelvis ring with two 7.3 mm partially threaded short cannulated self-tapping screws (lengths: 90 mm for S1 and 65 mm for S2) without washers.

Set 2:

- Group FT w/: Fixation of the posterior pelvis ring with two 7.3 mm fully threaded short cannulated self-tapping screws (lengths: 90 mm for S1 and 65 mm for S2) and washers (diameter 13.0 mm, thickness 6.6 mm).
- Group FT w/o: Fixation of the posterior pelvis ring with two 7.3 mm fully threaded short cannulated self-tapping screws (lengths: 90 mm for S1 and 65 mm for S2) without washers.

Set 3:

- Group TS w/: Fixation of the posterior pelvis ring with two 7.3 mm fully threaded long transsacral cannulated self-tapping screws (lengths: 175 mm for S1 and 160 mm for S2) and washers (diameter 13.0 mm, thickness 6.6 mm).
- Group TS w/o: Fixation of the posterior pelvis ring with two 7.3 mm fully threaded long transsacral cannulated self-tapping screws (lengths: 175 mm for S1 and 160 mm for S2) without washers.

Equal numbers of three right and three left pelvis sides were utilized in each group (n = 6). The surgical treatment was performed according to the AO principles [22] as follows. After reduction of the SI joints, the self-tapping screws were positioned over a previously placed guidewires perpendicular to the SI joint, entirely within the artificial bone, avoiding any perforations. The insertion of the guidewires was performed with a custom aiming guide, allowing a standardized placement through a single attempt without any perforations or cortical disturbances. First, the S1 SI screw was positioned, followed by the S2 SI screw. The screws ranged to the midline of the sacral vertebra in groups PT w/, PT w/o, FT w/and FT w/o, and to the contralateral ilium cortex in groups TS w/and TS w/o, and were tightened according to the operator’s best judgment. Post instrumentation, true lateral, inlet and outlet x-rays were captured to verify the positioning of the inserted screws (Figure 1).

All screws and washers were made of stainless steel (316LVM) and the same manufacturer (DePuy Synthes, Zuchwil, Switzerland) provided all implants. The anterior pelvis ring was cut by sawing the pelvic ramus at the middle of the shaft on the ipsilateral and contralateral sides, as this reflects the injury pattern under investigation. A qualified surgeon operated all pelvises.

### 2.1. Biomechanical Testing

**Figure 2:**
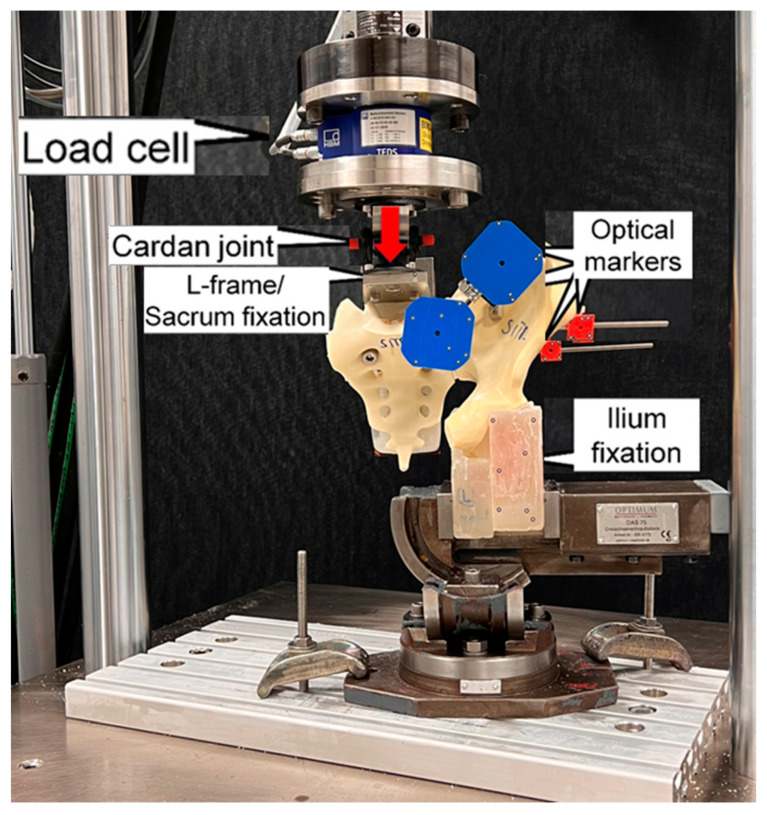
Setup with a specimen mounted for biomechanical testing. Vertical arrow indicates loading direction.

The biomechanical testing procedure and equipment was described in a previous study [23]. The system was equipped with a 5 kN/50 Nm load cell, operating at less than 1% accuracy within 10–100% loading capacity [24,25]. The setup with a specimen mounted for biomechanical testing is visualized in Figure 2. Each specimen was aligned and tested in an upright standing position. The ilium part of the examined anatomical side was fixed to the base of the machine using a vice, gripping the part of the ilium between the ramus and the border of the acetabulum. Two custom polymethylmethacrylate blocks were modelled for repeated use, ensuring a standardized clamping force applied by the vice on the clamped specimen region. Loading along the machine axis was applied to the sacrum with 41 mm anterior offset relative to the posterior-superior S1 endplate aspect, generating the required bending moment around the medio-lateral axis as calculated from a previous inverse-dynamic gait analysis [19]. A custom steel plate with L-profile was used for this purpose. The sacrum was attached to the vertically oriented side of the plate with two screws inserted through its fourth foramen. The horizontal part of the plate was attached to the machine actuator through a cardan joint. Four retro-reflective marker sets were attached to the sacrum, the iliac crest and both SI screws for optical motion tracking.

The loading protocol was adopted identically from a previous study [26].

### 2.2. Data Acquisition & Analysis

Machine data in terms axial displacement and axial load were continuously obtained from the machine transducer and the load cell throughout testing, respectively. On this basis, construct stiffness was evaluated from the ascending load–displacement curve of the initial quasi-static ramp.

The three-dimensional coordinates of all optical markers were continuously recorded identically as described before [26]. Based on the motion tracking data, the following parameters of interest were evaluated: (1) gap angle, defined as the combined angular displacement of the sacrum in coronal and horizontal plane relative to the ilium; (2) flexion, defined as the angular flexion displacement of the sacrum relative to the ilium; (3) screw tilt ilium, defined as the angular displacement of the S1 screw relative to the ilium and (4) screw tip cutout, defined as the translational displacement of the S1 screw tip relative to the sacrum in the plane perpendicular to the screw axis. The outcome measures were evaluated at three time points of cyclic testing after 1000, 2000, and 3000 cycles, under maximal load of the respective cycle and relative to the beginning of the cyclic test. The latter number represented the highest rounded number of cycles when none of the specimens had failed. Reaching 5° gap angle was arbitrary set as a clinically relevant failure criterion, and the corresponding number of cycles until its fulfillment under maximal load of the corresponding cycle—defined as cycles to failure—was calculated together with the corresponding maximal load, defined as load at failure.

Statistical analysis was performed with SPSS software (v.27, IBM SPSS, Armonk, NY, USA). Normality of data distribution was screened and proved with Shapiro–Wilk test. Significant differences between the group pairs within each set (using same screw design) regarding construct stiffness, cycles to failure and load at failure were detected with Independent-Samples t-test. General Linear Model Repeated Measures test was conducted to identify significant differences between the group pairs within each set with regard to the parameters of interest evaluated over the time points after 1000, 2000, and 3000 test cycles. Level of significance was set at 0.05 for all statistical tests.

## 3. Results

Construct stiffness was 47.0 ± 11.1 N/mm (mean value ± standard deviation, SD) in group PT w/and 29.4 ± 7.4 N/mm in group PT w/o (set 1), 33.1 ± 8.2 N/mm in group FT w/and 27.7 ± 9.7 N/mm in group FT w/o (set 2), and 31.9 ± 8.4 N/mm in group TS w/and 47.5 ± 18.7 N/mm in group TS w/o (set 3). Group PT w/was associated with significantly higher stiffness compared to group PT w/o, *p* = 0.012. No significant differences were detected between the other two pairs of groups with and without a washer in set 2 (FT w/versus FT w/o) and set 3 (TS w/versus TS w/o), *p* ≥ 0.110.

The outcome measures analyzed over the three time points after 1000, 2000, and 3000 cycles are summarized in Table 1. Gap angle and screw tilt ilium were associated with significantly higher values in group FT w/o compared to its paired group FT w/, *p* ≤ 0.028. Furthermore, flexion trended towards higher values in group FT w/o compared to group FT w/, *p* = 0.083, with no further significant differences between the group pairs within each set with regard to the two parameters of interest, *p* ≥ 0.121.

**Figure 3:**
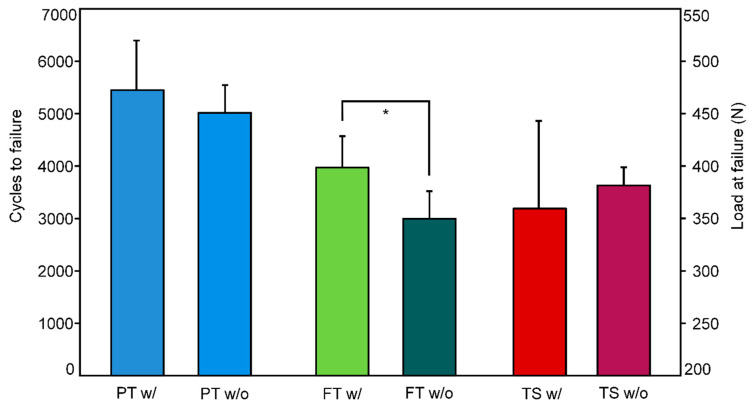
Cycles to failure (left abscissa) and corresponding load at failure (right abscissa), presented for each separate group in terms of mean value and SD. Star indicates significant differences between the groups.

Cycles to failure and load at failure were 5452 ± 944/694.3 ± 47.2 N in group PT w/and 5016 ± 531/450.8 ± 26.6 N in group PT w/o (set 1), 3972 ± 600/398.6 ± 30.0 N in group FT w/and 2993 ± 527/349.7 ± 26.4 N in group FT w/o (set 2), and 3189 ± 1674/359.5 ± 83.7 N in group TS w/and 3630 ± 348/381.5 ± 17.4 N in group TS w/o (set 3), respectively. Group FT w/was associated with significantly higher values for both of these parameters of interest compared to group FT w/o, *p* = 0.026 (Figure 3). No significant differences were detected between the other two pairs of groups with and without a washer in set 1 (PT w/versus PT w/o) and set 3 (TS w/versus TS w/o), *p* ≥ 0.359. Failure modes for all fixation methods were expressed by a fracture of the ilium between the polymethylmethacrylate ilium fixation and the S2 SI screw.

## 4. Discussion

The goal of this study was to assess the stability of standard S1-S2 SI screw fixations, with and without using a washer, across different screw designs. It was hypothesized that in good bone quality the omission of the washer would result in equivalent biomechanical stability. The following two main important points can be identified:

1. Partially threaded short and fully threaded long transsacral screws demonstrated equivalent biomechanical stability under dynamic loading disregarding whether a washer was used or not.
2. Omission of the washer led to a significantly diminished biomechanical stability under dynamic loading with fully threaded short screws.

The surgical therapy of the qualified groups in the presented study is regularly used in clinical practice [27,28,29]. However, previous work could not reveal significant differences between the different types of SI screw fixation [27,30,31]. In contrast, the experimental setting in the current investigation seems to be relevant to detect even the smallest differences in biomechanical behavior between the groups, such as the choice of washer inclusion.

The routine use of a washer for fixation of the posterior pelvis ring seems to be at the discretion of the treating surgeon and is scarcely discussed in the current literature. Therefore, an investigation is clearly difficult due to the lack of studies. A recent review and expert opinion concerning transsacral screw fixation of posterior pelvis ring injuries does not cover this topic in any manner and only devotes a single sentence to it: “We routinely utilize a washer if there is fracture displacement” [6,32].

A comparable study, using fully threaded 7.3 mm sacroiliac S1 screws, described washer penetration in the iliac bone [19]. In our study, no penetration of the screw heads or washers into the cortices of the bone model occurred in any group. According to the operator’s judgement, the screws with and without washer could be tightened with comparable strength. This seems to be reflected in the results with comparable initial construct stiffness between the fixations using the same screw design with and without a washer, yet with significantly higher stiffness in group PT w/versus group PT w/o.

Characteristically, the initial stiffness of partially threaded screws with a washer was higher, although this supposed advantage seems to be eliminated during dynamic loading, at least to the point where the screw head eventually breaks into the cortex. With fully threaded short screws on the other hand, it was demonstrated that although the initial stiffness was comparable, the advantages of using a washer only become apparent during dynamic loading.

Washers are known as safeguards against screw intrusion as well as a technique to retain compressive force after screw intrusion [6]. Furthermore, they are a standard component in the iliosacral screw placement recommendations from the AO [22]. Yet, there is little data on over-compression of the SI joints in the literature. Most authors merely describe that this should be avoided [32,33,34]. It has further been reported as a disadvantage that fixation of posterior SI joint dislocations with transiliac sacral bars, including over-compression of the ilium, may lead to damage to the sacral nerves [35]. A different study regarding this topic recommends cannulated 7.3 mm partially threaded screws with a washer, however, fully threaded washerless screws may be preferred to avoid over-compression of a sacral fracture in case of comminution [34].

A transiliac fixator for treatment of comminuted sacrum fractures has the potential for higher stiffness with a lower risk of over-compression [36]. However, judging the quality of reduction is difficult and there is a risk of over-compression with nerve injury when a sacral fracture is present [37].

The amount of applied compression seems to be left to the experience of the surgeon. The judgement of the treating operator should be utilized to avoid over-compression of the fracture of the posterior pelvis ring [32]. The same competence should be attributed with regard to screw intrusion. In addition, this previous investigation found that there were no significant differences between the treatment groups in terms of gap angle and screw tilt in the ilium, suggesting that in young patients, the exclusion of the washer seems to be feasible. However, the current study demonstrated significantly higher values for these two parameters of interest (gap angle and screw tilt in ilium) in group FT w/o compared to group FT w/. In addition, the number of cycles to failure and load at failure were significantly higher in the latter versus the former group. In our test setup, the symphysis was not fixed, as it might happen in a clinical situation [3].

A biomechanical study investigating cement augmentation of SI screws concluded that bilateral bone implant anchorage was the significantly more important factor concerning fracture displacement than the actual number of inserted screws [37]. It has further been stated that longer screws achieve higher stability than shorter screws [3].

### Strength & Limitations

The obvious limitation of this investigation is the chosen artificial bone model. This makes the simulation of ligament injuries of the pelvis, as well as screw intrusion, difficult. Though, as this is an innovative study with a debatable approach, for which there is limited data available and an unpredictable outcome, the authors agreed to the artificial specimens as a first step approach. Following a successful first artificial bone study, it is ethically acceptable to proceed with a cadaver investigation. Further, the investigated injury was purposely chosen to provide an absolute unstable situation representing the worst-case scenario. Therefore, the missing ligaments in the chosen specimens could be disregarded in the performed test series.

Furthermore, artificial pelvises allow standardized experimental groups, which can overpower innumerable differences in the bone quality of human cadavers and are also more cost-effective [38,39,40,41]. Specifically, when investigating the pelvis, artificial bone specimens have been repeatedly and effectively used in many biomechanical studies [31,38,42,43,44]. Moreover, the accessibility of cadavers is not only limited, but the specimens are also costly, both of which can influence the sample size of biomechanical research. It is already recognized that the sample sizes used in earlier publications are in general small [45]. While the sample size in this investigation was moderately small, it is equivalent to comparable biomechanical studies investigating posterior pelvis fixation techniques [31,42,43,44,46]. Missing torque data on the tightening force of the screws with and without washers is another limitation. However, since all screws were implanted by the same experienced surgeon and there was no screw head or washer penetration into the bone, it can be assumed that all screws were tightened with a comparable strength. Finally, the witnessed failure modes, fracturing of the ilium in between the ilium fixation and S2 SI screw, seem to be far from clinically relevancy, especially for young patients. Therefore, the bones used may not have been able to properly simulate certain screw loosening effects, e.g., cut-through of the proximal screw part into the cortex. Nevertheless, the knowledge gained can serve as a basis for further research on different bone models or cadaveric bones.

## 5. Conclusions

From a biomechanical perspective, a washer could be optional when using partially threaded short or fully threaded long transsacral S1-S2 screws for treatment of posterior pelvis ring injuries in young trauma patients. Yet, the omission of the washer in fully threaded short screws could lead to a significantly diminished biomechanical stability. Additional biomechanical cadaveric studies should be initiated for a reliable interpretation of washerless fixations and their potential for the therapy of the injured posterior pelvis ring.

## Figures and Tables

**Table 1:** Outcome measures evaluated after 1000, 2000 and 3000 cycles, presented for each group in terms of mean value and SD, together with *p*-values from the statistical evaluation over cycles and between the group pairs within each set.

Parameter	Group	Cycles			*p*-Value Groups
		1000	2000	3000	
**Gap angle (°)**	PT w/	0.50 (0.39)	0.84 (0.66)	1.19 (0.87)	0.751
	PT w/o	0.47 (0.20)	0.92 (0.36)	1.45 (0.56)	
	FT w/	0.28 (0.30)	0.74 (0.50)	1.30 (0.65)	0.028
	FT w/o	1.31 (0.49)	3.52 (2.04)	6.46 (4.47)	
	TS w/	2.68 (2.33)	3.67 (2.58)	4.84 (2.76)	0.236
	TS w/o	1.26 (1.07)	2.26 (1.43)	3.23 (1.67)	
	***p*-value** **over cycles**	≤0.038			
**Flexion (°)**	PT w/	1.09 (1.31)	2.47 (2.27)	3.22 (2.61)	0.973
	PT w/o	0.96 (0.30)	2.10 (0.71)	3.81 (0.77)	
	FT w/	0.87 (0.27)	1.80 (0.58)	2.90 (0.90)	0.083
	FT w/o	3.32 (2.62)	6.03 (5.03)	9.91 (6.80)	
	TS w/	4.47 (3.24)	6.65 (4.13)	10.63 (5.94)	0.132
	TS w/o	2.23 (1.77)	3.93 (2.62)	5.62 (3.53)	
	***p*-value** **over cycles**	≤0.042			
**Screw tilt ilium (°)**	PT w/	0.68 (0.57)	1.38 (0.88)	1.92 (1.02)	0.452
	PT w/o	0.71 (0.13)	1.48 (0.27)	2.59 (0.27)	
	FT w/	0.50 (0.23)	1.13 (0.48)	1.87 (0.75)	0.011
	FT w/o	2.12 (1.05)	5.25 (2.64)	9.34 (4.47)	
	TS w/	3.17 (2.24)	4.73 (2.78)	7.26 (3.87)	0.232
	TS w/o	1.88 (1.25)	3.30 (1.82)	4.75 (2.29)	
	***p*-value** **over cycles**	≤0.011			
**Screw tip cutout (mm)**	PT w/	0.12 (0.07)	0.23 (0.18)	0.28 (0.22)	0.139
	PT w/o	0.09 (0.07)	0.23 (0.15)	0.60 (0.09)	
	FT w/	0.17 (0.09)	0.35 (0.08)	0.37 (0.22)	0.121
	FT w/o	0.54 (0.48)	0.98 (1.02)	1.76 (1.27)	
	TS w/	0.57 (0.37)	1.33 (0.90)	2.96 (2.02)	0.742
	TS w/o	0.89 (1.05)	1.51 (1.29)	1.82 (1.96)	
	***p*-value** **over cycles**	≥0.216 (PT w/, FT/w, TS/w) ≤0.023 (PT w/o, FT w/o, TS w/o)			

PT w/and PT w/o: groups with partially threaded short screws with and without washers; FT w/and FT w/o: groups with fully threaded short screws with and without washers; TS w/and TS w/o: groups with fully threaded long transsacral screws with and without washers

## Data Availability

The datasets analyzed during this study are available from corresponding author upon reasonable request.
